# Ictal Epileptogenic Network Differences in Posterior Cingulate Epilepsy Subtypes Based on SEEG

**DOI:** 10.1002/cns.71192

**Published:** 2026-10-01

**Authors:** Zhaofen Yan, Yujiao Yang, Jing Wang, Qin Qin Deng, Minghui Wang, Huajun Yang, Jiali Pan, Jian Zhou, YuGuang Guan, Guoming Luan, Mengyang Wang

**Affiliations:** ^1^ Department of Neurology, Sanbo Hospital Capital Medical University Beijing China; ^2^ Department of Epilepsy Center, Sanbo Hospital Capital Medical University Beijing China

**Keywords:** epileptogenic network, node in‐strength, node out‐strength, PCC epilepsy, relative strength index, RSC epilepsy

## Abstract

**Aims:**

To compare differences in ictal propagation between epileptogenic networks between posterior cingulate cortex (PCC) epilepsy and retrosplenial cortex (RSC) epilepsy and to elucidate their network‐level pathophysiological characteristics.

**Methods:**

A total of 18 patients with confirmed PCC or RSC‐related epilepsy were enrolled in this study. We calculated directed functional connectivity across preictal, ictal onset, and propagation phases based on pairwise nonlinear regression (*h* ^2^ coefficient). Using graph theory and linear mixed‐effects models, we quantified network topology and dynamic propagation patterns of epileptogenic networks.

**Results:**

Neuropsychological tests showed significantly poorer visual recall in patients with RSC epilepsy (*p* = 0.030). During the propagation phase, the ipsilateral mesial temporal structures showed significantly greater node out‐strength than in‐strength, acting as a secondary driver of seizure spread (*p* = 0.004). The lateral temporal cortex acted as a signal receiver with dominant in‐strength (*p* = 0.015). RSC epilepsy also showed stronger directional propagation toward ipsilateral mesial temporal areas than PCC epilepsy (*p* = 0.022), whereas PCC epilepsy lacked consistent directional connectivity across key regions.

**Conclusion:**

RSC and PCC epilepsy display inherent differences in visuospatial memory performance and ictal network dynamics. The preferential propagation toward ipsilateral mesial temporal structures in RSC epilepsy may represent a potential electrophysiological correlate for distinguishing posterior cingulate epilepsy subtypes.

## Graphical abstract

Visual recall function was more severely impaired in patients with RSC epilepsy than in those with PCC epilepsy. During the propagation phase, ipsilateral mesial temporal structures exhibited a predominant “driver” role, whereas lateral temporal cortices functioned as “receivers.” RSC epilepsy also demonstrated stronger propagation strength toward mesial temporal regions than PCC epilepsy, which demonstrated no clear directional features.

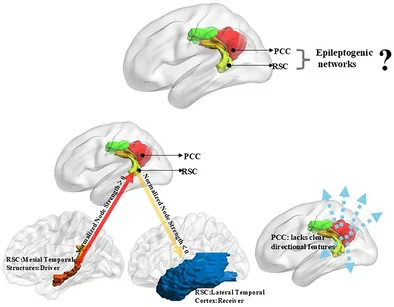

## 1 Introduction

Posterior cingulate epilepsy (PCE) is a clinically challenging form of drug‐resistant focal epilepsy. Precise localization of the epileptogenic zone remains difficult due to complex and variable ictal semiology. The posterior cingulate gyrus contains several functionally distinct subregions, among which the posterior cingulate cortex (PCC) and retrosplenial cortex (RSC) represent two major epileptogenic origins [1]. Clinically and electrophysiologically, seizures arising from PCC and RSC may present with overlapping manifestations including impaired consciousness, automatisms, complex motor symptoms, tonic seizures, and autonomic disturbances. However, clear symptomatic differences exist: automatisms are more frequent in RSC‐onset seizures, which also carry a longer duration of impaired consciousness [1, 2, 3]. These differences stem from the inherent divergences in cytoarchitecture, structural connectivity, and functional network organization between the two subregions.

From the perspective of anatomical and functional foundations, the PCC is predominantly composed of granular neurons with high neuronal density and abundant synaptic connections. Its core connectivity network is concentrated in regions such as the medial frontal lobe, precuneus, and temporoparietal neocortex, and it is extensively involved in motor control, somatosensory processing, and visual information integration [4, 5, 6]. In contrast, the RSC contains a higher proportion of agranular cells and exhibits relatively sparse neuronal distribution. A defining characteristic of the RSC is its dense projectional connectivity with the hippocampus, parahippocampal gyrus (PHG), and dorsolateral prefrontal cortex, which is significantly higher than that observed in the PCC [6], rendering it a core brain region for the storage, formation, and retrieval of spatial memory [7, 8]. Such differences in structural connectivity would theoretically lead to a specific divergence in the propagation patterns of epileptogenic networks during seizure onset between the two subtypes; however, this hypothesis has not yet been systematically validated in clinical studies.

Stereoelectroencephalography (SEEG) enables direct recording of dynamic changes in ictal electroencephalographic dynamics [9], providing a robust platform for analyzing epileptogenic network propagation [3, 10]. Yet the core driving nodes, detailed propagation pathways, and cognitive mechanisms remain poorly understood in these two epilepsy subtypes. Functional connectivity analysis combined with graph theory allows quantitative assessment of network topology [11] and identification of key driver and receiver nodes, making it a valuable tool for distinguishing epilepsy subtypes [12, 13, 14].

This study aimed to characterize directed functional connectivity from SEEG data and to combine graph theory with statistical models to address three goals: (1) to identify differences in strength and directionality of functional connectivity across seizure phases; (2) to determine whether the two subtypes follow distinct propagation pathways; and (3) to verify whether network topological features correlate with clinical and cognitive profiles. The findings of this study are expected to provide novel electrophysiological evidence supporting subtype differentiation of posterior cingulate epilepsy and to facilitate precise preoperative localization of the epileptogenic zone.

## 2 Methods

### 2.1 Patient Selection

This retrospective study analyzed patients with PCE subtypes who underwent SEEG examinations at the Epilepsy Center of Sanbo Brain Hospital, Capital Medical University, between 2012 and 2023.

**Inclusion Criteria**: (1) The seizure‐onset zone (SOZ) was confirmed by SEEG to be located in the PCC or RSC;(2) At least two complete habitual seizures were recorded during SEEG monitoring; (3) Patients underwent PCC resection or PCC/RSC radiofrequency thermocoagulation, with a postoperative follow‐up period exceeding 1 year and an Engel Class I outcome (seizure‐free).

**Exclusion Criteria**: (1) The core origin of the SOZ could not be clearly identified as PCC or RSC via SEEG confirmation; (2) Fewer than two complete epileptic seizures were recorded during SEEG monitoring; (3) Patients with coexisting neurological disorders or a history of severe traumatic brain injury were excluded (such conditions may interfere with the assessment of epileptogenic networks).

All patients were consecutively enrolled following unified inclusion and exclusion criteria. Each participant completed standard preoperative evaluations, including clinical history collection, neurological examination, video‐EEG monitoring, epilepsy‐specific MRI, PET, and neuropsychological testing with the Wechsler Memory Scale‐IV. According to individual anatomical features, all patients received hypothesis‐driven SEEG electrode implantation and long‐term intracranial monitoring to identify the SOZ and propagation pathways.

### 2.2 Ethical Considerations

The study protocol was approved by the Human Subject Ethics Committee of the Sanbo Brain Hospital, Capital Medical University (Ethics Code: 2025–050‐01). Given the retrospective nature of the study and the use of routinely collected clinical data, the requirement for individual informed consent was waived by the Institutional Review Board. This study was conducted in accordance with the principles of the Declaration of Helsinki.

### 2.3 SEEG Surgery and Recording Methods

Individualized electrode implantation plans were formulated based on each patient's clinical symptoms, electrophysiological features, and anatomical structures. The electrode coverage strategy focused on: (1) core hypothesized regions: PCC and RSC, and (2) propagation regions: ipsilateral mesial temporal structures (with primary attention to the hippocampus), the neocortical temporal lobe, precuneus, neocortical parietal lobe, and selected contralateral homologous brain regions to ensure adequate spatial sampling.

A Nicolet 256‐channel system was used to record the EEG signals. A high‐pass filter of 0.16 Hz was applied, with a sampling rate of ≥ 256 Hz. Preoperative MRI was co‐registered with the MNI‐152 standard brain atlas (1.0 mm isotropic voxels) using SPM 12 software (revision 7771; https://www.fil.ion.ucl.ac.uk/spm/software/spm12/) to standardize regions of interest into the MNI space. Frequency color‐encoded visualization was performed using MRIcroGL software (version 1.2.20220720; https://www.nitrc.org/projects/mricrogl). Postoperative CT scans were co‐registered to preoperative three‐dimensional T1‐weighted sequences using in‐house‐developed GARDEL software for precise localization of SEEG electrode contact positions (Figure 1A).

**FIGURE 1:**
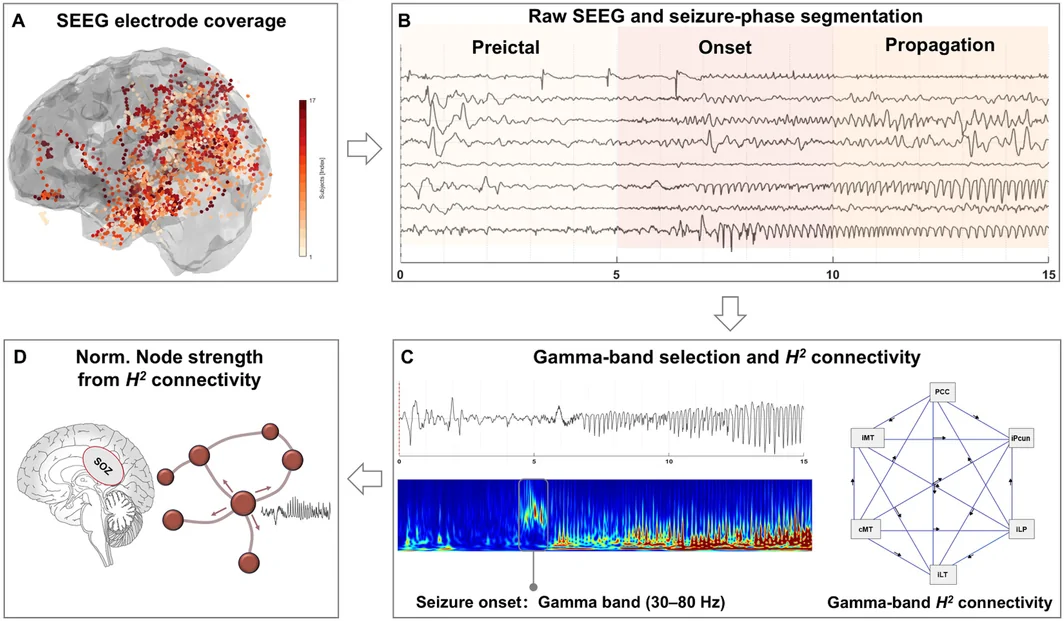
Example of SEEG exploration and signal analysis in Posterior Cingulate Epilepsy. (A) All SEEG electrode placements in 18 patients with a lateral view to show the anatomical extent of implantations in the posterior cingulate cortex and extra posterior cingulate cortex areas. (B) Illustration of the Ictal SEEG and seizure‐phase segmentation for functional connectivity (FC) analysis. (C) Functional connectivity among raw signals was filtered in the frequency bands: 30–80 Hz. Gamma band‐based *h* ^2^ analysis was applied to assess functional connectivity between the PCC and other brain regions (iMT, cMT, iLT, iLP, iPcun). (D) Node strength derived from *h* ^2^‐directed functional connectivity reveals epileptogenic network features identifying the PCC as the SOZ. cMT, contralateral mesial temporal; iLP, ipsilateral lateral parietal; iLT, ipsilateral lateral temporal; iMT, ipsilateral mesial temporal; iPcun, ipsilateral precuneus; PCC, posterior cingulate cortex; SOZ, seizure onset zone.

### 2.4 Epileptogenicity Index Analysis and Functional Connectivity

#### 2.4.1 Identification and Grouping of Epileptogenic Zones

First, inter‐rater reliability of anatomical localization for SEEG contacts (PCC vs. RSC) was assessed using Cohen's Kappa coefficient. All electrode contacts were independently classified by two experienced neurologists. The Kappa value was calculated based on a 2 × 2 contingency table. The inter‐rater reliability was substantial (Kappa = 0.85, 95% CI: 0.74–0.96), indicating high consistency in anatomical grouping. Then, the Epileptogenicity Index (EI) was calculated to evaluate the ability of brain regions to generate rapid discharges during epileptic seizures (with a maximum value of 1). Based on this, the regions were categorized as follows: epileptogenic zone (EZ, EI > 0.4), propagation zone (PZ, 0.2 ≤ EI ≤ 0.4), and non‐involved zone (NI, EI < 0.2) [10, 11]. SEEG electrode contacts were divided into two groups: (1) contacts located within the PCC; (2) contacts located within the RSC. A bipolar montage was constructed to minimize the effects of volume conduction.

#### 2.4.2 Time Intervals of Interest

For each seizure monitored via SEEG, three consecutive 5 s time windows were extracted for analysis: (1) Preictal phase: the 5 s prior to seizure onset (−5 to 0 s), serving as a baseline control; (2) Ictal onset phase: the 5 s following seizure onset (0 to 5 s), representing early seizure activity; (3) Propagation phase: 5 to 10 s after seizure onset, representing early seizure propagation. Seizure onset was defined as the time point at which the first clear, sustained, and evolving rhythmic discharge, distinct from background activity, appeared in at least one bipolar channel (Figure 1B).

#### 2.4.3 Functional Connectivity Calculation

To determine the frequency band for functional connectivity analysis, a time‐frequency analysis was first performed on the ictal onset period of all epileptic seizures. The results indicated that EEG activity at seizure onset was predominantly concentrated within the gamma band (30–80 Hz, Figure 1C), which aligns with previous studies demonstrating the role of high‐frequency oscillations in seizure initiation [15]. Based on this finding, we selected the 30–80 Hz frequency band to calculate functional connectivity.

Functional connectivity was quantified using the nonlinear correlation coefficient (*h* ^2^) embedded in AnyWave software. As an information theory‐based metric, *h* ^2^ can detect nonlinear relationships between electrophysiological signals. It exhibits directional asymmetry, with unequal bidirectional coupling strengths (*h* ^2^(*x* → *y*) ≠ *h* ^2^(*y* → *x*)), which helps identify the dominant signal propagation direction within the epileptogenic network.

Compared with Granger causality and phase‐based approaches, *h* ^2^ is better suited for analyzing non‐stationary ictal SEEG data. It reliably captures nonlinear neuronal interactions in the gamma band and overcomes the limitations of methods that only assess linear or phase‐locked coupling [16]. We calculated gamma‐band *h* ^2^ coefficients for all SEEG channel pairs using a 3 s sliding window with 1.5 s overlap to construct directed functional connectivity matrices. No threshold was applied to the raw *h* ^2^ values, ensuring retention of subtle but biologically valid functional connections. The resulting matrices were finally adopted for graph‐theoretical analysis.

#### 2.4.4 Computation of Graph Theory Metrics

The following graph theory metrics were calculated based on directed connectivity matrices to quantify the role of each SEEG channel within the epileptogenic network:

1. Node in‐strength: The sum of all *h* ^2^ values for connections directed toward the node, reflecting the node's capacity to receive information from the network.
2. Node out‐strength: The sum of all *h* ^2^ values for connections originating from the node to other nodes, reflecting the node's capacity to drive network activity.
3. Total node strength: The sum of node in‐strength and node out‐strength, representing the overall connectivity strength of the node within the network.
4. Average total node strength: The mean of total node strength across all channels within each time window, serving as a normalized reference for overall network connectivity strength.
5. Normalized node strength: Calculated as (Out‐strength—in‐strength)/Average total node strength. This metric quantifies the directional role of a node (i.e., driver versus receiver) while controlling for variations in the overall network connectivity across time windows and between patients [17]. For example, if a PCC node displays greater out‐strength than in‐strength, the PCC is considered to occupy a “driver” position [11] (Figure 1D).

### 2.5 Statistical Analysis

#### 2.5.1 General Information and Clinical Characteristics

Data were analyzed using SPSS software (version 26.0). Continuous variables conforming to normal distribution are presented as mean ± standard deviation and were compared using an independent samples *t*‐test. Non‐normally distributed continuous variables are expressed as median (range) and were compared using the Mann–Whitney *U*‐test. Categorical variables are presented as numbers (percentages). Given the small sample size, between‐group comparisons of all categorical variables were performed using Fisher's exact test. Statistical significance was set at *p* < 0.05.

#### 2.5.2 Linear Mixed‐Effects Model Analysis

To quantify the spatiotemporal dynamics of functional connectivity during seizure evolution, two Linear Mixed‐Effects Models (LMMs) were constructed to account for the repeated‐measures data structure and interindividual variability. The temporal dimension was divided into three seizure phases: preictal, ictal onset, and spread. The spatial dimensions included seven key brain regions: RSC, PCC, ipsilateral mesial temporal structures (iMT), contralateral mesial temporal structures (cMT), ipsilateral lateral temporal cortex (iLT), ipsilateral lateral parietal cortex (iLP), and ipsilateral precuneus (iPcun).

**I. Intra‐Group Temporal Dynamics Analysis**

To examine changes in connection strength during seizure propagation in RSC and PCC epilepsy, LMM was constructed separately for each group, as follows:

$$y_{\mathrm{ij}}=\beta_{0}+\beta_{1}\cdot {\text{Epoch}}_{j}+u_{i}+\varepsilon_{\mathrm{ij}}$$

where $y_{\mathrm{ij}}$ represents the connection strength of the target brain region for the i‐th patient during the j‐th seizure phase, *Epoch* (fixed effect) denotes the seizure phase, $u_{i}$ is the patient‐specific random intercept (random effect), and $\varepsilon_{\mathrm{ij}}$ represents the residual term. The parameters were estimated using restricted maximum likelihood (REML). For each target brain region, a Type III analysis of variance (ANOVA) was first conducted to test the significance of the main effect of seizure phase (α=0.05). If a significant main effect was observed, post hoc pairwise comparisons (ictal vs. preictal, spread vs. ictal) were performed using Tukey's correction for multiple comparisons.

**II. Inter‐Group Comparison of Connection Strength**

To compare output connection strength between RSC and PCC epilepsy across different target brain regions within each seizure phase, independent LMM were constructed for each seizure phase and each target brain region. The model was specified as follows:

$$y_{\mathrm{ik}}=\beta_{0}+\beta_{1}\cdot {\text{Group}}_{i}+u_{i}+\varepsilon_{\mathrm{ik}}$$

where *Group* (fixed effect) indicates the type of epileptic origin (RSC vs. PCC), and $u_{i}$ represents the patient‐specific random intercept. The regression coefficient ($\beta_{1}$), standard error (SE), and p‐value for the *Group* fixed effect were obtained. Estimated marginal means and their 95% confidence intervals (CI) were also computed for both groups to quantify the effect size of between‐group differences.

All statistical analyses were performed using the *fitlme and Anova functions in MATLAB R2020a*. Two‐tailed tests were applied, and p<0.05 were considered statistically significant. To account for multiple independent tests across different target brain regions, the Benjamini–Hochberg procedure was used to control the false discovery rate (FDR < 0.05) for the inter‐group comparison results.

## 3 Results

### 3.1 Clinical Data

A total of 18 patients (36 seizures) were enrolled in this study, including 8 cases of RSC epilepsy (all male, fulfilled the inclusion criteria) and 10 cases of PCC epilepsy (5 males and 5 females; Figure 1C). The overall mean age at surgery was 20.01 ± 8.41 years (range: 7.00–38.00 years), the mean age at epilepsy onset was 12.28 ± 5.69 years (range: 3.00–27.00 years), and the mean preoperative disease duration was 8.06 ± 6.62 years (range: 1.00–21.00 years).

No statistically significant differences were observed between the two groups in terms of age at surgery, age at epilepsy onset, interictal or ictal video‐EEG characteristics, imaging findings, surgical approach, or postoperative outcomes (all *p* > 0.05). However, the mean disease duration was shorter in the RSC epilepsy group (5.50 ± 4.07 years) than that in the PCC epilepsy group (10.10 ± 7.71 years) (*p* = 0.050), and a significant difference in sex distribution was observed between the groups (*p* = 0.040). Among patients who completed neuropsychological testing and had a total memory quotient above 70 points, five cases were in the RSC epilepsy group, and seven were in the PCC epilepsy group. The visual reproduction subscale score was significantly lower in the RSC epilepsy group (3.40 ± 5.64 points) compared to the PCC epilepsy group (10.29 ± 2.14 points) (*p* = 0.030). No statistically significant differences were found between the two groups in total memory quotient, paired‐associate learning, logical memory, or working memory (all *p* > 0.05; Table 1).

**TABLE 1:** Patients' demographic and clinical characteristics.

Variables	RSC	PCC	*p*
Demographic data			
Male/female	8/0	5/5	0.040
Age at surgery	19.13 ± 7.32	21.30 ± 9.46	0.371
Age at seizure onset	10.88 ± 4.88	13.63 ± 6.67	0.523
Duration (year)	5.50 ± 4.07	10.10 ± 7.71	0.050
Seizure characteristics (S.F)			
Behavior arrest	9/16	5/20	0.090
Bil. automotor	10/16	6/20	0.090
Ips. automotor	2/16	4/20	0.670
EEG (IID)			
Ips. T	2/8	1/10	0.559
Bil. T	1/8	2/10	1.000
Ips. F. T	1/8	2/10	1.000
Bil. T. O	3/8	4/10	1.000
No epileptiform discharges	1/8	1/10	1.000
EEG (ID)			
Diffuse	8/16	7/20	0.500
Ips. T. O	4/16	7/20	0.718
Ips. H	2/16	2/20	1.000
Ips. F. T	2/16	2/20	1.000
Ips. T	0/16	2/20	0.492
MRI			
No lesion	6/8	6/10	0.64
Lesion	2/8	4/10	0.64
Memory quotient	82.80 ± 32.21	104.75 ± 21.86	0.13
Visual reproduction	3.40 ± 5.64	10.29 ± 2.14	0.03
Verbal paired associates	6.80 ± 3.96	6.86 ± 3.58	0.11
Logical memory	7.40 ± 3.36	8.57 ± 2.57	0.25
Working memory	9.00 ± 3.54	11.57 ± 4.16	0.14
Surgery			
Resecting lesions	3/8	5/10	0.74
RF‐TC	5/8	5/10	0.43
Outcome (Engel class)			
I	6/8	8/10	1
II	2/8	2/10	1

*Note:* In addition, all memory assessment indicators derived from the WMS‐IV test in Table 1 are presented as scaled scores.

Abbreviations: Bil. automotor, Bilateral automotor; Bil. T, Bilateral temporal; Bil.T.O, Bilateral temporal occipital; ID, Ictal duration; IID, Interictal duration; Ips. automotor, Ipsilateral automotor; Ips. F.T, Ipsilateral frontal temporal; Ips. H, Ipsilateral hemisphere; Ips. T, Ipsilateral temporal; Ips. T.O, Ipsilateral temporal occipital; RF‐TC, Radiofrequency thermocoagulation; S.F, Seizure characteristics: Semiological features.

### 3.2 Temporal Characteristics of Intra‐Group Differences in Connection Strength

To investigate within‐group directional connectivity between PCC epilepsy or RSC epilepsy and the ipsilateral mesial temporal structures, temporal neocortex, precuneus, and parietal neocortex across different seizure phases, directional characteristics were analyzed. Compared to the preictal and ictal onset phases, RSC epilepsy in the propagation phase showed significantly higher node out‐strength than node in‐strength toward ipsilateral mesial temporal structures (*p* = 0.004), suggesting that ipsilateral mesial temporal structures predominantly acted as “drivers” of network information during this period. Conversely, the temporal neocortex exhibited significantly higher node in‐strength than node out‐strength (*p* = 0.015), displaying typical “receiver” characteristics.

In contrast, PCC epilepsy showed no statistically significant differences in any of the seven key brain regions (all *p* > 0.05), indicating that the epileptogenic network in PCC epilepsy may lack distinct directional features (Figure 2).

**FIGURE 2:**
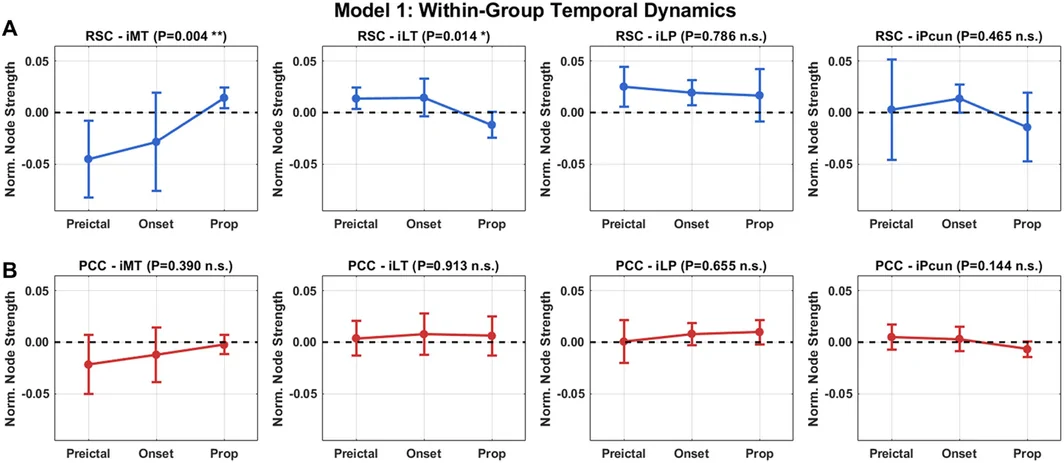
During epileptic seizures, the changes in the normalized node strength between corresponding brain regions in RSC epilepsy and PCC epilepsy. (A) RSC epilepsy group: During the propagation phase, the normalized relative strength of the IMT node was significantly greater than 0 (*p* = 0.0040), indicating that this node acts as a “driver.” Meanwhile, the normalized relative strength of the ILT node was significantly less than 0 (*p* = 0.0140), indicating that this node acts as a “receiver.” No significant differences in node strength were observed in other brain regions. (B) PCC epilepsy group: Across all analyzed brain regions, no significant differences in normalized relative strength were found (all *p* > 0.05).

### 3.3 Spatiotemporal Characteristics of Inter‐Group Differences in Connection Strength

This study further examined differences in relative connection strength between RSC epilepsy and PCC epilepsy across seven key brain regions during the same seizure phases (preictal, ictal, and spread). No statistically significant inter‐group differences were observed in any brain region during the preictal or ictal onset phases. However, during the spread phase, a significant difference in relative strength emerged between RSC epilepsy and PCC epilepsy with respect to the ipsilateral mesial temporal structures (*p* = 0.022) (Figure 3). This finding suggests that RSC epilepsy preferentially recruits the ipsilateral mesial temporal structures.

**FIGURE 3:**
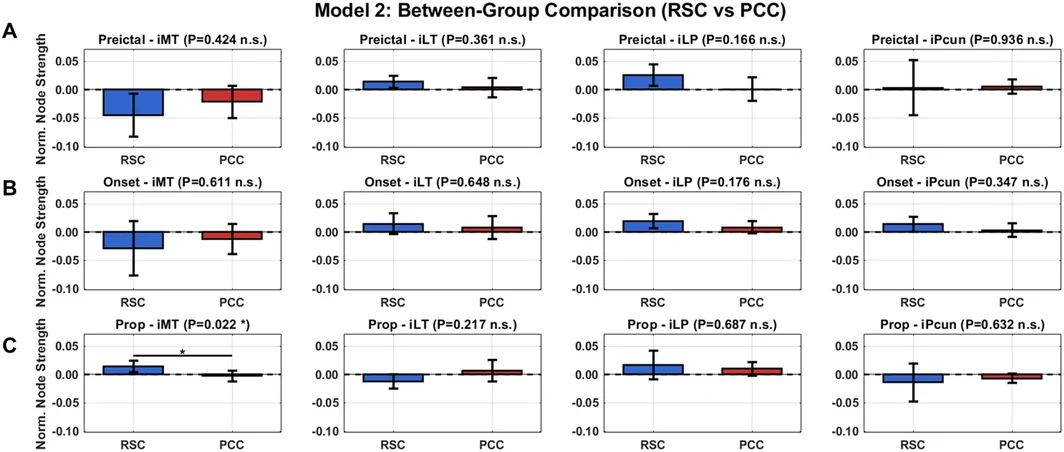
Spatiotemporal characteristics of inter‐group differences in connectivity strength. (A, B) No statistically significant differences in normalized node strength were observed between the two groups across any brain regions during either the pre‐ictal or the ictal onset phase. (C) During the propagation phase, the RSC epileptogenic group exhibited a statistically significant increase (*p* = 0.022, *p* < 0.05) in normalized node strength to the ipsilateral mesial temporal structures compared to the PCC group during the propagation phase.

### 3.4 Differences in Connectivity Strength Between RSC and PCC Epileptogenic Networks and Bilateral Hippocampal Structures

To further investigate potential differences in connectivity between the epileptogenic networks of RSC epilepsy and PCC epilepsy, and the bilateral hippocampal structures, an inter‐group comparison was conducted involving 18 patients from both cohorts who had electrodes implanted in the ipsilateral hippocampal structures. The results showed that the relative connectivity strength of the ipsilateral hippocampal structures was significantly higher in the RSC epilepsy group during the spread phase (*p* = 0.021, *p* < 0.05) (Figure 4A,B). In an additional analysis of six seizures from three patients in each group with electrodes implanted in the contralateral hippocampal structures, no significant difference in connectivity strength was observed between the two groups in the contralateral hippocampal structures (*p* = 0.211, *p* > 0.05) (Figure 4C,D). These findings further suggest that the epileptogenic network in RSC epilepsy exhibits a specific propagation pathway involving the ipsilateral hippocampal structures, a feature not observed in PCC epilepsy.

**FIGURE 4:**
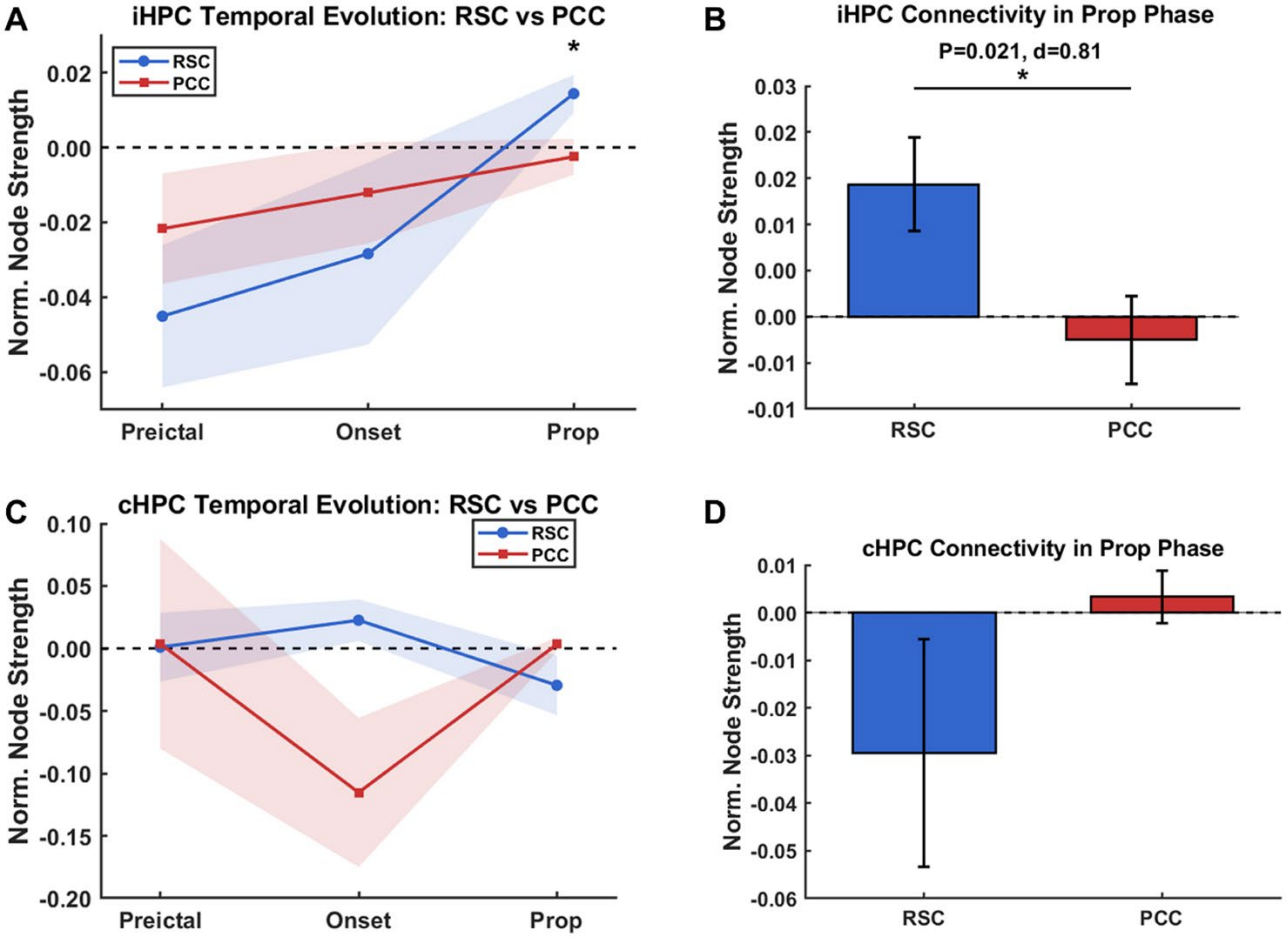
Analysis of connectivity strength between RSC and PCC epileptogenic networks and bilateral hippocampal structures across different seizure phases. (A) No statistically significant differences were observed between the two groups during the preictal and onset phases. During the propagation phase, however, connectivity strength between the RSC and the ipsilateral hippocampus was significantly higher than that between the PCC and the ipsilateral hippocampus. (B) In the propagation phase, a statistically significant difference was identified between the connectivity strength of the RSC and that of the PCC with the ipsilateral hippocampus (*p* = 0.021). (C, D) Across all seizure phases, no statistically significant differences (*p* > 0.05)were found in the connectivity strength between either the RSC or PCC and the contralateral hippocampal structure.

## 4 Discussion

This study is the first to analyze the electrophysiological connectivity characteristics of posterior cingulate epilepsy subtypes during seizures using SEEG, and to examine dynamic changes in functional connectivity during ictal activity. The findings revealed the following: (1) Compared to the preictal and ictal onset phases, RSC epilepsy during the spread phase exhibited significantly higher node out‐strength than in‐strength toward ipsilateral mesial temporal structures, whereas the temporal neocortex showed significantly higher node in‐strength than out‐strength. These results suggest that, within the epileptogenic network of RSC epilepsy, inward connectivity is enhanced in the ipsilateral mesial temporal structures, whereas outward connectivity is strengthened in the temporal neocortex. (2) Compared with PCC epilepsy, RSC epilepsy preferentially recruits the ipsilateral mesial temporal structures during the spread phase. (3) The specific network topological feature characterized by enhanced connectivity to the ipsilateral hippocampal structures during the spread phase of RSC epilepsy is intrinsically associated with patients' impaired visual–spatial memory function. This network characteristic could mirror different electrophysiological profiles between RSC and PCC epilepsy, offering tentative theoretical hints for future investigations on subtype distinction and preoperative localization.

**I. Epileptogenic Network of RSC Epilepsy: Phase‐Specific Directed Propagation Patterns and Mechanisms**

This study identified both “phase‐dependency” and “laterality‐specificity” in the propagation of the epileptogenic network in RSC epilepsy. During the preictal and ictal onset phases, no significant differences were observed in connectivity between the RSC and the mesial temporal structures, temporal neocortex, or parietal cortex. However, during the spread phase, the ipsilateral mesial temporal structures exhibited significantly higher node out‐strength than in‐strength, whereas the temporal neocortex showed higher node in‐strength than out‐strength. These results indicate that the epileptogenic network in RSC epilepsy specifically propagates toward the ipsilateral mesial temporal structures during the spread phase, a process closely associated with seizure initiation in the RSC. In contrast, the temporal neocortex appears to function as a critical hub influenced by long‐range connections within the RSC epileptogenic network. Collectively, these findings suggest that during the spread phase, the ipsilateral mesial temporal structures act as “drivers,” whereas the temporal neocortex serves as the primary “receiver” in the RSC epileptogenic network.

Specific Propagation of the Epileptogenic Network in RSC Epilepsy and the Intrinsic Neuroanatomical and Functional Connectivity Characteristics of the RSC. Previous primate studies have identified bidirectional connections between the RSC and hippocampus, PHG, and anterior thalamic nuclei. However, the RSC receives substantially more direct afferent inputs from the hippocampal CA1 region and subiculum than indirect projections to the hippocampus via the entorhinal cortex and subiculum complex [18]. Through the integration of modulation techniques and electrophysiological recordings, prior studies have further characterized the functional coupling between the RSC and the hippocampus. Studies have shown that hippocampal inactivation influences neuronal firing patterns in the RSC, while existing data indicate that RSC inactivation alters the firing properties of hippocampal place cells [19] and disrupts experience‐dependent plasticity in the RSC [20]. The specific propagation pathway toward the ipsilateral hippocampus identified in the present study is consistent with these established findings.

Swanson et al. [21, 22] conducted electrophysiological studies in mouse models and demonstrated that the hippocampus and RSC form a weakly coupled excitable system capable of bidirectional modulation, with the RSC potentially acting as a core node in the epileptogenic network that actively influences downstream cortical regions. In the present study, the enhanced outward connectivity of the ipsilateral mesial temporal structures during the spread phase of RSC epilepsy reflects this “core node–driver” mechanism in the pathological state of epilepsy. These brain regions may switch from their physiological function in memory encoding to network nodes that amplify and spread epileptic discharges, which could be linked to aberrant activation of downstream temporal neocortical areas [23].

Notably, the “receiver” role of the temporal neocortex observed in the epileptogenic network of RSC epilepsy in the present study differs from previous findings, suggesting that the “RSC is predominantly linked to the hippocampus, whereas the PCC exhibits closer connections with the temporal neocortex” [3]. This discrepancy may stem from the “cascade activation” effect within the epileptogenic network [24, 25]; although direct connectivity between RSC and the temporal neocortex is weaker than that of the PCC, it is possible that the ipsilateral mesial temporal structures serve as an “intermediate driver,” thereby forming an indirect propagation pathway of “RSC‐ipsilateral hippocampus–temporal neocortex.” Through this pathway, the temporal neocortex may ultimately act as the final receiver and amplifier of aberrant epileptic signals [26]. These findings align with earlier research on “cascade activation within the default mode network” during RSC epileptic seizures, which indicates that the RSC, as a core node of the default mode network, drives abnormal activity in related regions such as the ipsilateral hippocampus and temporal neocortex through cascade activation. Furthermore, cascade activation has been shown to correlate with impaired visuospatial memory function [27].

Collectively, these observations expand the current understanding of the RSC epileptogenic network, suggesting that its propagation is not confined to the mesial temporal circuit but can extend to the temporal neocortex through cascade‐mediated network effects.

**II. Epileptogenic Network in PCC Epilepsy: Diffuse Connectivity and Non‐Directional Propagation Characteristics**

In contrast to RSC epilepsy, PCC epilepsy showed no significant differences between node in‐strength and out‐strength across all seizure phases and key brain regions, including the mesial temporal structures, temporal neocortex, and parietal cortex, suggesting that its epileptogenic network lacks clear directional features.

The posterior cingulate cortex is involved in multiple visual processing functions such as object and emotion recognition of visually presented stimuli, stimulus localization, perception of body position, and observation of hand movements [28]. Furthermore, functional MRI and electrophysiological studies in non‐human primates have demonstrated that the dorsal PCC participates in monitoring decision‐making performance [29], while the ventral PCC exhibits spatially specific responses during visual recognition or cued recall of people, places, and objects [30]. Research also indicates that the dorsal PCC plays an important role in linking long‐term memory with the decision‐making process [31] and acts as a hub connecting the default mode network and the salience network [32].

In the present study, the diffuse connectivity observed in the epileptogenic network of PCC epilepsy may be related to the diversity and variability of its seizure propagation pathways. Within the default mode network, functional connectivity between the angular gyrus and the posterior cingulate cortex is relatively stronger than connectivity involving many other brain regions. Furthermore, functional connectivity analysis in cingulate epilepsy has identified significantly enhanced connectivity between the left angular gyrus and the left posterior cingulate cortex [33]. Previous studies have also reported increased connectivity between the left superior frontal gyrus and the left precuneus in patients with temporal lobe epilepsy, whereas connectivity between the left hippocampus and the left posterior cingulate cortex, as well as between the left hippocampus and the left precuneus, is reduced [34]. However, systematic investigations of the epileptogenic network in cingulate epilepsy remain limited. In the present study, no dominant propagation pathway was identified within the epileptogenic network of PCC epilepsy. During seizures, abnormal electrical activity originating in the PCC does not propagate directionally along a dominant anatomical pathway, but instead simultaneously activates multiple brain regions through widespread network connections, resulting in an epileptogenic network that lacks clearly defined “driver” and “receiver” roles. This aligns with the clinical manifestations of PCC epilepsy: compared to the specific impairment of visual reproduction function often observed in RSC epilepsy, seizure symptoms in PCC epilepsy are more diffuse and lack clear localizing signs.

**III. Clinical Significance and Research Implications of Network Propagation Differences**

The divergent propagation patterns identified between RSC and PCC epileptogenic networks may represent unique electrophysiological heterogeneity across subtypes, providing preliminary theoretical references for subsequent clinical subtype validation research. With respect to epileptogenic zone localization, particularly in patients with posterior cingulate epilepsy who lack clear structural abnormalities on imaging, the specific electrophysiological features of RSC epilepsy—namely, “increased outward connectivity from the ipsilateral mesial temporal structures and increased inward connectivity to the temporal neocortex during the spread phase” may serve as distinctive biomarkers for RSC epilepsy, effectively distinguishing it from the diffuse connectivity pattern observed in PCC epilepsy.

Furthermore, the network propagation characteristics identified in this study were significantly correlated with neuropsychological assessment results. This study found that visual reproduction function was significantly impaired in patients with RSC epilepsy. Functional MRI studies suggest that the RSC serves as a key hub connecting the cortical default mode network with the medial temporal memory system, thereby playing a central role in cognitive processes, such as spatial navigation, episodic memory retrieval [35], and self‐referential thinking [36]. Moreover, the bidirectional connections among the RSC, anterior thalamic nuclei, and hippocampus constitute a limbic memory circuit that is critical for visual memory retrieval [37] and spatial representation reconstruction [38]. The directional spread of ictal electrical activity within this network may alter the physiological function of memory‐related circuits [34, 39], which may correlate with the visuospatial memory deficits observed in patients with RSC epilepsy. In contrast, while PCC epilepsy is also involved in visual information integration, the posterior cingulate cortex may mediate the variability in behavioral choices in response to environmental information, which may explain the absence of similar spatial memory deficits [40]. Collectively, the association between epileptogenic network propagation patterns and clinical phenotypes identified in this study provides an important framework for strategies aimed at preserving cognitive function.

Due to the nonspecific clinical symptoms and rapid propagation pathways characteristic of posterior cingulate epilepsy, accurate localization of the epileptogenic zone remains challenging, with long‐term postoperative seizure‐free rates reported at approximately 50% [41]. Given the high heterogeneity of the patient population, individualized treatment strategies are particularly important. In patients with RSC epilepsy, when the node out‐strength of the ipsilateral hippocampus during the spread phase significantly exceeds its in‐strength, the epileptogenic zone should encompass not only the RSC but also potential involvement of the ipsilateral hippocampal structures as downstream nodes recruited during the spread phase (rather than independent seizure onset sites). Conversely, in PCC epilepsy, owing to the absence of clearly defined directional propagation pathways, surgical planning should focus on the core subregions of the PCC and their directly adjacent nodes, while avoiding excessive resection that may increase the risk of postoperative cognitive impairment.

## 5 Study Limitations and Future Directions

Some limitations should be acknowledged. First, the sample size was relatively small, and all patients in the RSC epilepsy group were male, which may limit the generalizability of the findings, particularly with respect to sex‐related differences. Future studies should expand multicenter cohorts and incorporate sex‐matched subgroup analyses to further validate the robustness and generalizability of these conclusions. Second, anatomical connectivity techniques, such as diffusion tensor imaging, were not incorporated in this study, thereby precluding direct quantification of fiber connection strength between the RSC/PCC and the bilateral mesial temporal structures. Future research integrating SEEG with DTI could facilitate the construction of combined “anatomical‐functional” network models, which may help clarify the relationship between structural connectivity and functional propagation. Third, the spatial coverage of stereotactic electrode placement was limited in this study, with electrode placement primarily focused on the temporoparietal cortex and not extending to distal regions such as the prefrontal cortex and anterior cingulate cortex. Future investigations should include targeted electrode placement in the prefrontal cortex and anterior cingulate regions to more comprehensively characterize the full network connectivity of PCC‐related epileptogenic circuits.

## 6 Conclusion

RSC and PCC epilepsy demonstrate fundamentally distinct propagation patterns within epileptogenic networks during seizures. RSC epilepsy, with seizure onset originating in the RSC, exhibits directional spread toward the ipsilateral mesial temporal structures (particularly the hippocampal formation), which is associated with impaired visuospatial memory. In contrast, PCC epilepsy shows a relatively diffuse and non‐directional propagation pathway. Collectively, these findings provide crucial electrophysiological evidence to support precise clinical diagnosis, individualized surgical decision‐making, and the preservation of cognitive function in patients with posterior cingulate epilepsy.

## Funding

This work was supported by Ministry of Science and Technology of China (2022ZD0205000), National Key Research and Development Plan (2022YFC2503801), and the National Natural Science Foundation of China (32020103009).

## Conflicts of Interest

We report no conflicts of interest related to this work. We confirm that we have read the Journal's position on issues involved in ethical publication and affirm that this report is consistent with those guidelines.

## Data Availability

The data that support the findings of this study are available on request from the corresponding author. The data are not publicly available due to privacy or ethical restrictions.
